# Retrorectal tumors over 25 years: Surgical strategies and long-term outcomes from a high-volume tertiary center

**DOI:** 10.1097/MD.0000000000048012

**Published:** 2026-03-20

**Authors:** Fadime Kutluk, Sefa Ergün, Süleyman Demiryas, Murat Süphan Ertürk, Abdullah Kağan Zengin, Mehmet Sinan Çarkman, Engin Hatipoğlu, Rauf Hamid, Sabri Şirolu, Emrecan Sari

**Affiliations:** a Department of General Surgery, Florence Nightingale Hospital, Istanbul, Türkiye; b Department of General Surgery, Cerrahpaşa Faculty of Medicine, Istanbul University–Cerrahpaşa, Istanbul, Türkiye; c Department of Radiology, Sungurlu State Hospital, Çorum, Türkiye; d Department of Radiology, Cerrahpaşa Faculty of Medicine, Istanbul University–Cerrahpaşa, Istanbul, Türkiye.

**Keywords:** colorectal surgery, postoperative complications, preoperative biopsy, presacral mass, retrorectal tumors, surgical approach, tailgut cyst

## Abstract

Retrorectal tumors are rare and heterogeneous lesions that present significant diagnostic and surgical challenges. Owing to their low incidence, consensus on optimal management strategies is lacking, and large-scale outcome data remain limited. This study aimed to present a large, long-term, single-center experience with retrorectal tumors, focusing on surgical strategies, recurrence outcomes, and the clinical relevance of preoperative biopsy decisions. This retrospective cohort study included 58 patients who underwent surgical treatment for retrorectal tumors between 2000 and 2025. A total of 61 patients were initially enrolled; 3 were censored at their last known contact in the Kaplan–Meier survival analysis. Accordingly, descriptive and comparative analyses were performed on the remaining 58 patients with complete follow-up. Clinical data, including demographics, imaging modalities, surgical approach, histopathological diagnosis, and follow-up outcomes, were collected and analyzed. The surgical approach was determined according to the tumor’s relationship to the third sacral vertebra. Statistical analyses included Kaplan–Meier survival estimation and appropriate comparative tests to evaluate postoperative outcomes. The cohort had a mean age of 46.8 ± 14.7 years, with a marked female predominance (81%). All patients underwent preoperative cross-sectional imaging, and preoperative biopsy was performed in 12% of cases. A posterior surgical approach was employed in 91% of patients. Tailgut cysts were the most frequent histopathological subtype (46.6%). No statistically significant association was observed between preoperative biopsy and postoperative complications (*P* > .05). During long-term follow-up, local recurrence occurred in 2 patients (3.4%). Although not statistically significant, a trend toward increased postoperative complications was observed in patients who underwent preoperative biopsy (*P* = .067). Although rare, retrorectal tumors can be managed effectively with accurate diagnosis, individualized surgical planning, and vigilant long-term surveillance. Preoperative biopsy should not be performed routinely and should be considered on a case-by-case basis, given its potential complication risk and limited diagnostic yield. This 25-year, single-center experience provides valuable insights into the multidisciplinary management of retrorectal tumors and supports evidence-based clinical decision-making in this anatomically complex region.

## 1. Introduction

Retrorectal tumors constitute a rare and heterogeneous group of lesions arising within the presacral space between the propria rectal fascia and the presacral fascia. They represent a highly heterogeneous group in terms of etiology, embryology, histology, and clinical presentation. In developed countries, the incidence is reported to be between 1 in 40,000 and 60,000 per year, with the majority of cases occurring in middle-aged women.^[1]^

Retrorectal tumors are often asymptomatic and are typically identified incidentally. In symptomatic cases, patients may present with nonspecific complaints such as abdominal pain, constipation, rectal bleeding, or urinary symptoms.^[2]^

A variety of diagnostic modalities can be employed, including digital rectal examination, flexible sigmoidoscopy, transrectal ultrasonography, computed tomography (CT), and magnetic resonance imaging (MRI). Histopathologically, retrorectal tumors are classified into congenital, neurogenic, osseous, inflammatory, and miscellaneous groups. Although the majority are benign, they carry a risk of malignant transformation, with reported rates ranging from 2% to 13% in the literature.^[3]^

When planning surgical treatment, the tumor’s anatomical relationship with the third sacral vertebra (S3) and potential invasion into adjacent structures must be carefully assessed. Therapeutic decisions (such as the selection of anterior vs posterior surgical approach, the preference for open or laparoscopic technique, and the potential need for neoadjuvant therapy) should be made by a multidisciplinary team.^[4]^

In this study, we retrospectively analyzed the clinical, radiological, and histopathological data of 58 patients with retrorectal tumors who underwent surgical treatment at our institution between 2000 and 2025. Despite growing awareness, surgical outcomes and recurrence risk factors remain under-reported, underscoring the need for large-scale retrospective analyses. Therefore, this study aimed to evaluate the long-term oncologic and surgical outcomes of retrorectal tumors within an anatomy-based framework, focusing on recurrence-free survival (RFS), complication risks, and the clinical implications of preoperative biopsy. By presenting one of the largest single-center cohorts with a 25-year follow-up, this study provides robust evidence to refine management strategies and guide selective biopsy decision-making.

## 2. Materials and methods

This retrospective cohort study initially included 61 patients who underwent surgery with a preoperative diagnosis of retrorectal tumor at a single tertiary care center (Department of General Surgery, Cerrahpaşa Medical Faculty, Istanbul University–Cerrahpaşa) between 2000 and 2025. Three patients were censored at their last known contact in the Kaplan–Meier survival analysis; therefore, descriptive and comparative analyses were based on 58 patients with complete follow-up data. Variables analyzed included demographic data (age, sex), clinical parameters (presenting symptoms, symptom duration, incidental diagnosis), radiological findings (imaging modality, tumor size, anatomical relationship to the S3), surgical characteristics (approach type, presence of combined or complete resection), postoperative outcomes (hospital stay, complications, recurrence status, histopathological diagnosis, and tumor size), and follow-up information (duration and recurrence). The current clinical status of each patient was obtained via telephone interviews. This retrospective study was approved by the Institutional Ethics Committee of Istanbul University–Cerrahpaşa, Cerrahpaşa Faculty of Medicine (Date: March 15, 2024, No: 1058472) and conducted in accordance with the Declaration of Helsinki. Due to the retrospective nature of the study, the requirement for written informed consent was waived by the ethics committee.

### 2.1. Surgical approach

**Figure 1.**
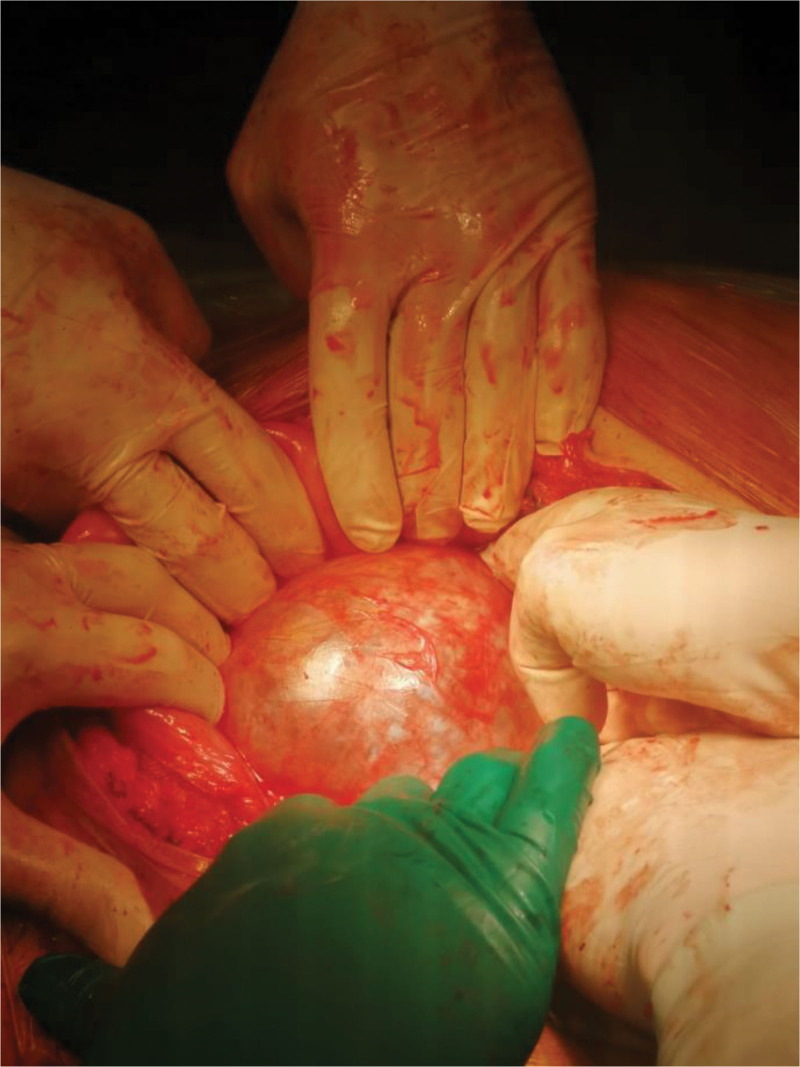
Intraoperative view showing the anterior approach via a lower midline laparotomy. The retrorectal cyst lies between the posterior vaginal wall and the anterior surface of the sacrum. The rectum is gently retracted posteriorly to expose the lesion for safe dissection along the presacral fascia.

The choice of surgical approach was determined by a multidisciplinary team based on the tumor’s relationship to the S3, proximity to adjacent anatomical structures (such as the sacrum, pelvic sidewalls, and urogenital organs), and its potential for malignancy. Tumors located below the level of S3 were managed via a posterior approach, while those above S3 were treated using an anterior (abdominal) approach (Fig. 1).

**Figure 2.**
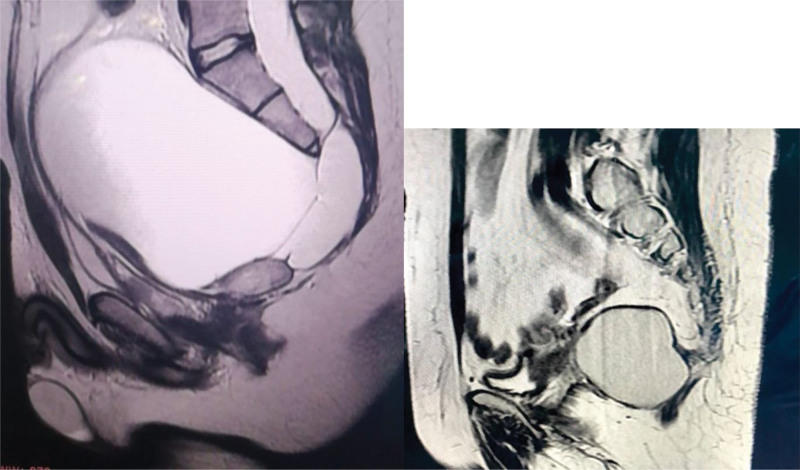
T2-weighted MRI showing a well-defined cystic retrorectal lesion (arrow) located between the rectum and the sacrum. MRI = magnetic resonance imaging.

All anterior resections were performed via open surgery. After mobilization of the sigmoid colon, dissection was performed along embryological planes anterior to the sacral promontory, preserving pelvic vessels, the mesorectum, ureters, and autonomic nerves. The tumor was excised from the space between the anterior mesorectum and posterior presacral fascia (Fig. 2).

**Figure 3.**
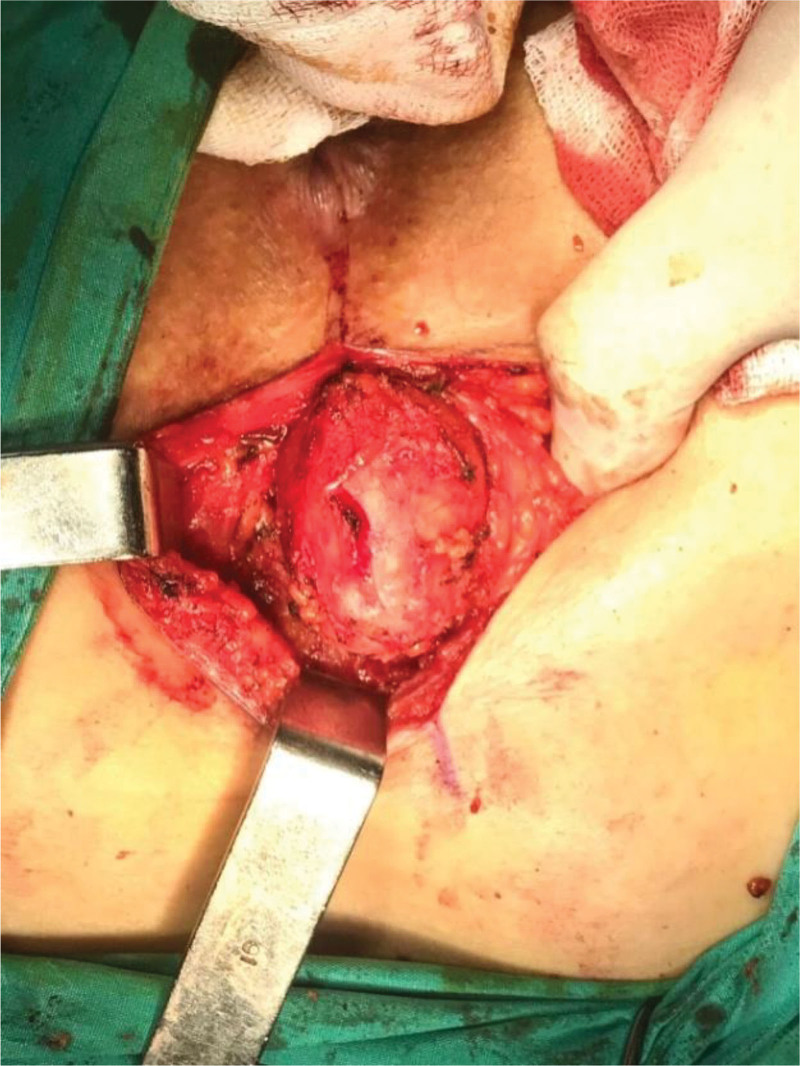
Posterior (paracoccygeal) approach performed in the jackknife position. Intraoperative photo demonstrates the cystic lesion being carefully dissected from the presacral fascia and coccyx. The gluteal muscles are retracted laterally to provide adequate exposure of the retrorectal space.

Posterior approaches (Kraske or transperineal parasagittal techniques) were performed with the patient in the jackknife position. Access to the retrorectal space was achieved by transecting the anococcygeal ligament. When necessary, bimanual palpation was used intraoperatively to delineate tumor borders and the rectal wall. The tumor was excised with the capsule intact. All anatomical layers were closed sequentially, and suction drains were placed in the excision cavity (Fig. 3).

### 2.2. Statistical analysis

Descriptive statistics included mean, standard deviation, median, minimum, maximum, frequency, and percentage values. The distribution of variables was assessed using the Kolmogorov–Smirnov and Shapiro–Wilk tests. For continuous variables with normal distribution, the independent samples *t* test was used; for non-normally distributed continuous variables, the Mann–Whitney *U* test was applied. Categorical variables were compared using the chi-square test or Fisher exact test when the chi-square assumptions were not met. Correlations between variables were analyzed using Spearman’s rank correlation coefficient. All statistical analyses were performed using SPSS version 27.0 (IBM Corp, Armonk). Statistical significance was defined as a *P*-value < .05. Due to the small number of events, multivariate analysis could not be performed for identifying independent predictors of complications or recurrence. Survival analysis included all 61 patients (3 censored). Descriptive and comparative analyses were based on 58 patients with complete follow-up data. According to Kaplan–Meier survival estimation, the 5-year RFS rate was calculated as 98%, with 2 recurrence events observed during long-term follow-up. Survival estimates were calculated for all 61 patients, including 3 censored cases, to ensure that loss to follow-up did not introduce any potential bias into the survival curve. The mean age of the patients was 46.8 ± 14.7 years, with a range of 18 to 78 years. Of the cohort, 47 patients (81%) were female, and 11 (19%) were male (Table 1). The majority of tumors (93%) were detected incidentally during imaging performed for unrelated complaints. Among symptomatic patients, the most common complaints were nonspecific abdominal pain and abdominal fullness (62%). The mean tumor size assessed by preoperative imaging was 5.8 ± 2.9 cm (median: 5 cm; range: 0.9–17 cm). Postoperative pathological analysis revealed that 95% of the lesions (n = 55) were benign and 5% (n = 3) were malignant. In terms of surgical approach, a posterior approach was performed in 91% of cases (n = 53), an anterior (abdominal) approach in 3% (n = 2), and a combined anterior–posterior approach in 5% (n = 3). The mean length of hospital stay was 9.5 ± 3.5 days. The postoperative complication rate was 7%, consisting entirely of surgical site infections and small fluid collections (abscesses) in 2 patients. All complications were classified as grade II according to the Clavien–Dindo classification and were successfully managed with antibiotic therapy. No patient required reoperation (Table 2).

**Table 1:** Patient demographics and clinical characteristics.

	Minimum–maximum	Median	Median ± SD/n (%)
Age	18.0–78.0	45.5	46.8 ± 14.7
Sex			
Female			47 (81%)
Male			11 (19%)
Incidental diagnosis			
No			4 (7%)
Yes			54 (93%)
Duration until diagnosis (mo)	2.0–64.0	12.0	19.6 ± 14.1
Maximum preoperative diameter (cm)	0.9–17.0	5.0	5.8 ± 2.9
Preoperative radiological imaging			
MRI			26 (45%)
CT			23 (40%)
CT + MRI			7 (12%)
MRI + CT + endoanal ultrasound (EAUS)			2 (3%)
Lesion location relative to the S3 vertebra			
Below the S3 vertebra			50 (86%)
Above the S3 vertebra			4 (7%)
Extending both above and below the S3 vertebra			4 (7%)
Preoperative endoscopy			
(−)			39 (67%)
(+)			19 (33%)
Surgical approach			
Posterior			53 (91%)
Anterior + posterior			3 (5%)
Anterior			2 (3%)
Combined resection			
(−)			52 (90%)
(+)			6 (10%)
Complete resection			
(−)			0 (0%)
(+)			58 (100%)

CT = computed tomography, MRI = magnetic resonance imaging, S3 = third sacral vertebra, SD = standard deviation.

**Table 2:** Surgical treatment and outcomes.

Surgical resection performed	58 (100%)
Surgical approach (n = 58)	
Posterior approach	53 (91%)
Anterior approach	2 (3%)
Combined approach	3 (5%)
Additional bone resection (coccygectomy) (n = 58)	6 (10.3%)
Postoperative complication (n = 58)	4 (7%)
Clavien–Dindo I	0 (0%)
Clavien–Dindo II	4 (7%)
Clavien–Dindo III	0 (0%)
Postoperative mortality (n = 58)	0 (0%)
Local recurrence	2 (3.4%)

The most frequently observed histopathological diagnosis was tailgut cyst, found in 27 patients (46.6%), followed by epidermoid cysts in 13 patients (22.4%) and mature cystic teratomas in 4 patients (6.9%). Other less common lesions included gastrointestinal stromal tumors and schwannomas, each detected in 3 patients. Enteric cystic hamartomas and hindgut embryonal cysts were observed in 2 patients each. In addition, rare entities such as ependymoma, hemangiopericytoma, chordoma, and neuroendocrine tumor (NET) arising in the wall of a tailgut cyst were each identified in 1 patient (Table 3).

**Table 3:** Histopathological findings.

Histopathological findings	n (%)
Benign tumors	46 (79.3%)
Tailgut cyst	27 (46.6%)
Epidermoid cyst	13 (22.4%)
Teratoma	4 (6.9%)
Embryonal cyst of the hindgut	1 (1.7%)
Enteric cystic hamartoma	1 (1.7%)
Borderline tumor	
Gastrointestinal stromal tumor (GIST)	3 (5.1%)
Schwannoma	3 (5.1%)
Hemangiopericytoma	1 (1.7%)
Malignant tumors	3 (5.2%)
Neuroendocrine tumor	1 (1.7%)
Ependymoma	1 (1.7%)
Chordoma	1 (1.7%)

## 3. Discussion

Retrorectal tumors are rare and heterogeneous lesions that continue to pose significant diagnostic and therapeutic challenges. Owing to their low incidence, large single- or multicenter series remain limited, and standardized management strategies are not fully established. In this context, our 25-year, single-center experience contributes to the existing literature by summarizing diagnostic approaches, histopathological distribution, and long-term outcomes of surgically treated cases within an anatomy-based framework.

In this study, the clinical, radiological, and histopathological data of 58 patients who underwent surgery for retrorectal tumors between 2000 and 2025 were retrospectively analyzed. The median age of the patients was 45 years, and 83.3% were female. This demographic distribution aligns with existing literature, which suggests that retrorectal tumors are more frequently observed in women.^[5]^

Most lesions in our cohort were detected incidentally; among symptomatic patients, nonspecific abdominal pain and constipation predominated. All patients underwent cross-sectional imaging (CT/MRI), consistent with current practice that regards these modalities as the diagnostic mainstay for retrorectal masses (Table 4). Only 12% underwent preoperative biopsy. Although the overall comparison did not reach conventional statistical significance, the observed difference in postoperative complications (29% vs 4%; *P* = .067) represents a clinically relevant trend and supports a selective rather than routine biopsy policy – particularly when imaging and surgical anatomy already indicate safe, en bloc excision. Patients with shorter symptom duration were more likely to undergo biopsy (*P* = .008), possibly reflecting a tendency to sample more recently discovered or acutely symptomatic lesions (Table 5).

**Table 4:** Postoperative outcomes and follow-up data.

Variable	Categories	n (%)	Median (range)	Median ± SD/n (%)
Complication	No	54 (93%)		
	Yes	4 (7%)		
Lesion	Primary	54 (93%)		
	Recurrent	4 (7%)		
Postoperative histopathological diagnosis	Benign	55 (95%)		
	Malignant	3 (5.2%)		
Recurrence during follow-up	No	56 (97%)		
	Yes	2 (3.4%)		
Preoperative histopathology (biopsy)	Not performed	51 (88%)		
	Performed	7 (12%)		
Length of hospital stay (d)			5.0–20.0	9.5 ± 3.5
Duration of follow-up (mo)			0.0–190.0	74.4 ± 40.8

“Recurrent lesion” refers to patients previously operated at other institutions. “Recurrence during follow-up” refers to tumor regrowth after primary surgery at our center.

“Primary lesion” refers to cases operated for the first time; not to be confused with incidental detection.

SD = standard deviation.

**Table 5:** Comparative analysis of clinical outcomes between patients with and without preoperative biopsy.

Variable	No biopsy (n = 51)	With biopsy (n = 7)	*P*-value (test)
Age (yr)	47.3 ± 14.6 (46.0 median)	42.8 ± 16.1 (42.5 median)	.484 (*t*)
Sex (F/M)	43/8 (84/16%)	4/3 (57/43%)	.117 (χ^2^)
Incidental diagnosis (yes/no)	47/4 (92/8%)	7/0 (100/0%)	1.000 (χ^2^)
Duration of symptoms (mo)	21.1 ± 14.4 (18.0 median)	8.9 ± 4.1 (12.0 median)	.008 (*m*)
Tumor diameter (cm)	5.8 ± 3.0 (5.0 median)	5.6 ± 2.6 (5.0 median)	.924 (*m*)
Preoperative endoscopy (+/−)	16/35 (31/69%)	3/4 (43/57%)	.544 (χ^2^)
Combined resection (+/−)	6/45 (12/88%)	0/7 (0/100%)	1.000 (χ^2^)
Complication (+/−)	2/49 (4/96%)	2/5 (29/71%)	.067 (χ^2^)
Primary/recurrent lesion	47/4 (92/8%)	7/0 (100/0%)	1.000 (χ^2^)
Histopathological diagnosis (benign/malignant)	48/3 (94/6%)	7/0 (100/0%)	1.000 (χ^2^)
Recurrence (+/−)	2/49 (4/96%)	0/7 (0/100%)	1.000 (χ^2^)
Hospital stay (d)	9.6 ± 3.6 (9.0 median)	9.3 ± 2.8 (8.0 median)	.922 (*m*)

*P*-value: independent samples *t* test (*t*), Mann–Whitney *U* test (*m*), or chi-square test (χ^2^) as appropriate.

**Figure 4.**
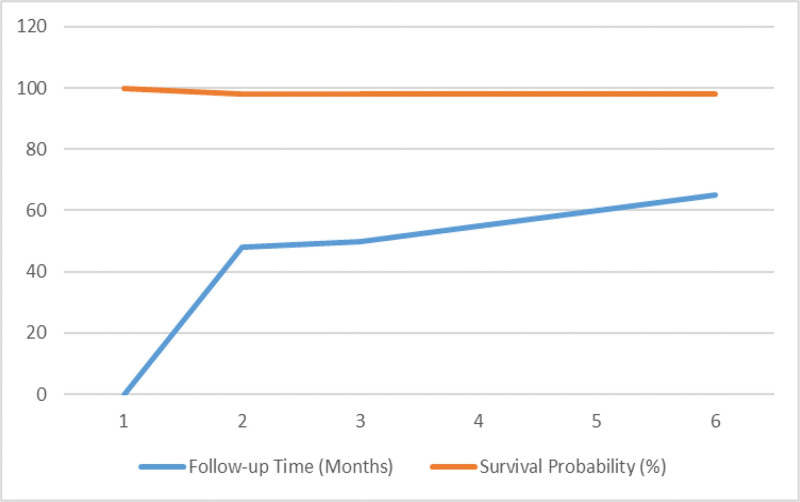
Kaplan–Meier curve demonstrating recurrence-free survival following surgical resection of retrorectal tumors. Tick marks indicate censored cases (n = 3). Two recurrence events were observed during follow-up. Numbers at risk are shown below the curve at 12-month intervals.

In terms of surgical approach, 91% of the patients underwent posterior, 3% anterior, and 5% combined (anterior–posterior) resection. The majority of the tumors were benign in nature, with a malignancy rate of 5.2%. Four patients presented with recurrent lesions (2 previously operated at other centers and 2 with recurrence during follow-up at our institution), highlighting the importance of distinguishing between recurrence types in outcome interpretation. During long-term follow-up, tumor recurrence was observed in only 2 patients (3.4%). The 5-year RFS rate of 98%, as shown in the Kaplan–Meier analysis, supports the long-term efficacy of surgical resection (Fig. 4). One underwent reoperation via the posterior approach, while the other declined surgical intervention and was managed with close surveillance. The malignancy rate identified in our study (5.2%) was lower than that reported by Baek et al (13.8%), Fechner et al, and Li et al (19.4%).^[2,5,6]^ However, it aligns with the 8% malignancy rate reported by Broccard et al.^[7]^ The most frequently observed histopathological diagnosis was tailgut cyst, which is consistent with findings from Baek and Messick, who also reported it as the most common lesion type.^[5]^ Epidermoid cysts were identified as the second most frequent histopathological diagnosis in our cohort. The findings of our study are largely consistent with previous reports in the literature.

The average patient age and the predominance of females parallel prior studies, indicating that retrorectal tumors are more common in women, typically between the ages of 30 and 60.^[8,9]^ Our rates of CT and MRI usage align with the established understanding that these imaging modalities are the gold standard for diagnosing retrorectal lesions.^[10,11]^ The higher rate of infections and complications observed in patients who underwent preoperative biopsy supports existing warnings in the literature regarding the infection risk associated with biopsy of retrorectal masses.^[12,13]^

Regarding surgical approach, our practice is consistent with the literature suggesting that the posterior approach is appropriate for benign lesions located below the S3 vertebra, whereas an anterior approach is preferred for lesions suspected to be malignant or located above S3.^[5,14]^ From a histopathological perspective, the finding that tailgut cysts were the most frequently identified type of retrorectal lesion in our cohort, with a malignancy rate of 5.2%, is consistent with the results reported in previous large-scale series.^[15–17]^ In our series, 1 patient with a previously resected tailgut cyst developed a recurrent lesion that was histopathologically diagnosed as a high-grade NET (G3 NET).^[14,18,19]^ This rare finding underscores the malignant potential of congenital retrorectal lesions and highlights the importance of complete excision during initial surgery. The case also exemplifies the need for careful preoperative imaging and postoperative surveillance, particularly for cystic lesions with irregular walls or solid components. These observations align with prior reports emphasizing that early and complete surgical excision remains the most effective strategy to prevent recurrence and malignant progression.^[17,18]^

In summary, our 25-year experience demonstrates that an anatomy-based surgical approach ensures safe and complete excision of retrorectal tumors, resulting in excellent long-term outcomes and very low recurrence rates. This observation aligns with recent multicenter analyses emphasizing the limited diagnostic yield and potential risk of tumor dissemination associated with preoperative biopsy in this rare disease group. The high-grade NET arising from a tailgut cyst in our cohort further illustrates the potential for malignant transformation, reinforcing the need for complete excision and long-term vigilance.

## 4. Limitations

Our study has several limitations, including its single-center nature and the lack of multivariate analysis due to the small number of recurrence and complication events. Nevertheless, the study provides one of the largest long-term, single-center datasets to date and contributes valuable insights into the optimal surgical management of retrorectal tumors. Future multicenter studies employing standardized imaging and pathological protocols are warranted to validate these findings and refine surgical decision-making in this challenging field. Building on these limitations, future research should focus on establishing multicenter registries integrating standardized radiological, histopathological, and surgical data. Incorporating molecular and genetic profiling may help identify biologically aggressive subgroups and refine individualized management strategies. Moreover, advances in AI-based image analysis could enhance noninvasive prediction of histological subtypes and risk stratification in retrorectal tumor surgery.

## 5. Conclusion

This 25-year, single-center study confirms that retrorectal tumors, though rare and heterogeneous, can be safely and effectively managed with individualized surgical strategies and long-term follow-up. Preoperative biopsy should not be routinely performed and must be considered selectively, given its potential complication risk and limited diagnostic yield in some cases. Given the size and duration of this single-center cohort, our study serves as one of the most comprehensive investigations into the long-term outcomes of surgically treated retrorectal tumors to date.

## Acknowledgments

The authors thank Prof M. Süphan Ertürk for their valuable contributions to the study design and scientific review. All individuals named in the acknowledgments have provided permission to be listed.

## Author contributions

**Conceptualization:** Abdullah Kağan Zengin, Mehmet Sinan Çarkman.

**Funding acquisition:** Sefa Ergün, Sabri Şirolu.

**Formal analysis:** Fadime Kutluk.

**Methodology:** Fadime Kutluk, Abdullah Kağan Zengin, Engin Hatipoğlu.

**Project administration:** Fadime Kutluk, Sefa Ergün, Süleyman Demiryas, Murat Süphan Ertürk, Mehmet Sinan Çarkman, Sabri Şirolu.

**Supervision:** Sefa Ergün, Süleyman Demiryas, Murat Süphan Ertürk, Abdullah Kağan Zengin.

**Resources:** Süleyman Demiryas.

**Software:** Mehmet Sinan Çarkman, Engin Hatipoğlu.

**Validation:** Engin Hatipoğlu.

**Data curation:** Sabri Şirolu, Emrecan Sari.

**Visualization:** Sabri Şirolu, Emrecan Sari.

**Investigation:** Emrecan Sari.

**Writing – original draft:** Fadime Kutluk, Rauf Hamid.

**Writing – review & editing:** Fadime Kutluk.
